# Loss of 5-hydroxymethylcytosine induces chemotherapy resistance in hepatocellular carcinoma via the 5-hmC/PCAF/AKT axis

**DOI:** 10.1038/s41419-022-05406-3

**Published:** 2023-02-02

**Authors:** Xiao-Jun Guo, Xiao-Yong Huang, Xuan Yang, Jia-Cheng Lu, Chuan-Yuan Wei, Chao Gao, Yan-Zi Pei, Yi Chen, Qi-Man Sun, Jia-Bin Cai, Jian Zhou, Jia Fan, Ai-Wu Ke, Yujiang G. Shi, Ying-Hao Shen, Peng-Fei Zhang, Guo-Ming Shi, Guo-Huan Yang

**Affiliations:** 1 Department of Liver Surgery and Liver Transplantation, Liver Cancer Institute, Zhongshan Hospital of Fudan University, 136 Yi Xue Yuan Road, Shanghai, 200032 PR China; 2 Key Laboratory for Carcinogenesis and Cancer Invasion, Chinese Ministry of Education, Shanghai, 200032 PR China; 3 Department of General Surgery, Peking University Third Hospital, Beijing, PR China; 4 Shanghai Key Laboratory of Medical Epigenetics, Institutes of Biomedical Sciences, Fudan University, Shanghai, 200032 PR China; 5 Department of Medical Oncology, Zhongshan Hospital of Fudan University, Shanghai, 200032 PR China; 6 Cancer Center, Zhongshan Hospital of Fudan University, Shanghai, 200032 PR China; 7 Clinical Research Unit, Institute of Clinical Science, Zhongshan Hospital of Fudan University, Shanghai, 200032 PR China

**Keywords:** Chemotherapy, Cancer therapeutic resistance

## Abstract

Multidrug resistance is a major challenge in treating advanced hepatocellular carcinoma (HCC). Although recent studies have reported that the multidrug resistance phenotype is associated with abnormal DNA methylation in cancer cells, the epigenetic mechanism underlying multidrug resistance remains unknown. Here, we reported that the level of 5-hydroxymethylcytosine (5-hmC) in human HCC tissues was significantly lower than that in adjacent liver tissues, and reduced 5-hmC significantly correlated with malignant phenotypes, including poor differentiation and microvascular invasion; additionally, loss of 5-hmC was related to chemotherapy resistance in post-transplantation HCC patients. Further, the 5-hmC level was regulated by ten-eleven translocation 2 (TET2), and the reduction of TET2 in HCC contributes to chemotherapy resistance through histone acetyltransferase P300/CBP-associated factor (PCAF) inhibition and AKT signaling hyperactivation. In conclusion, loss of 5-hmC induces chemotherapy resistance through PCAF/AKT axis and is a promising chemosensitivity prediction biomarker and therapeutic target for HCC patients.

## Introduction

Hepatocellular carcinoma (HCC) is the third cause of death among 36 common malignancies [1]. Chemotherapy is an essential strategy for solid malignancies; however, EACH study (which used oxaliplatin plus Fluorouracil/Leucovorin versus Doxorubicin as palliative chemotherapy in late-stage HCC) did not meet its primary endpoint [2], which indicating advanced HCC patients hardly benefited from this method. Resistance to commonly used chemotherapeutic agents, such as 5-fluorouracil (5-FU) and oxaliplatin, is a significant obstacle to the treatment of HCC and thus contributes to its high mortality rate [3, 4]. A better understanding of the underlying mechanism of chemotherapy resistance and developing novel therapeutic approaches in HCC patients are urgently needed.

Abnormal DNA methylation at the 5-position of cytosine (5-mC) is an essential epigenetic feature of malignancies and plays a vital role in tumorigenesis and progression [5, 6]. 5-Hydroxymethylcytosine (5-hmC) is converted from 5-mC via a reaction catalyzed by ten-eleven translocation (TET) family dioxygenases (TET1-3) and regulates gene transcription along with a broad range of biological processes, including stemness, development, and pathogenesis [7, 8]. Loss of 5-hmC is a hallmark of malignancy [9–12] and was found in many solid tumors [5, 13]. Despite there is a slight decrease of TET1 and TET3 in HCC, 5-hmC levels are significantly reduced in HCC tumors than in adjacent normal tissue; further investigations revealed that the reduction of 5-hmC level in HCC is associated with hepatitis B virus (HBV) infection and TET2 deficiency [14, 15]. Besides, amending 5-hmC abundance could inhibit the expression of chemoresistance-related genes, which suggests that TET2-regulated 5-hmC reduction is related to chemotherapy resistance in HCC [16]. These findings provide clues about the role of 5-hmC in the development and progression of HCC; however, the exact mechanism of chemotherapy resistance remains largely undetermined.

Side population (SP) cells, as defined by Hoechst dye exclusion in flow cytometry, were firstly described by Goodell et al. [17] and share characteristics with cancer stem cells (CSCs) [18]. Abundant studies, ours included, have indicated that SP cells exhibited chemoresistance potential in various malignant tumors [19–22], which reminds us it is a feasible approach to investigate the 5-hmC level in SP cells for revealing chemoresistance mechanisms. In this study, we surprisingly found that TET2 and 5-hmC levels were reduced in SP cells, and there is a negative relationship between 5-hmC level and SP proportions in HCC, which set a clue that 5-hmC reduction induced by TET2 deletion may relate to chemoresistance in HCC.

Histone acetyltransferase P300/CBP-associated factor (PCAF) is a GCN5-related N-acetyltransferase family member found initially to inhibit cellular transformation [23]. Recent studies have demonstrated that PCAF modulates the expression of several oncogenes and tumor suppressors through the acetylation of histones or transcription factors, consequently participating in cancer progression [24–26]. Specifically, PCAF induces autophagy in HCC cells through inhibition of the Akt/mTOR pathway [27] and hyperactivation of AKT signaling, which plays an essential role in HCC resistance to chemotherapy agents, including 5-FU and oxaliplatin [28, 29]. Based on this, our present investigation revealed that TET2 and 5-hmC deficiency induces chemoresistance through PCAF reduction and AKT signaling activation in HCC patients. These findings solidify the role of TET2/5-hmC in chemotherapy resistance.

## Results

### Low level of 5-hmC correlated with a high proportion of SP cells and a poor prognosis in HCC

**Fig. 1:**
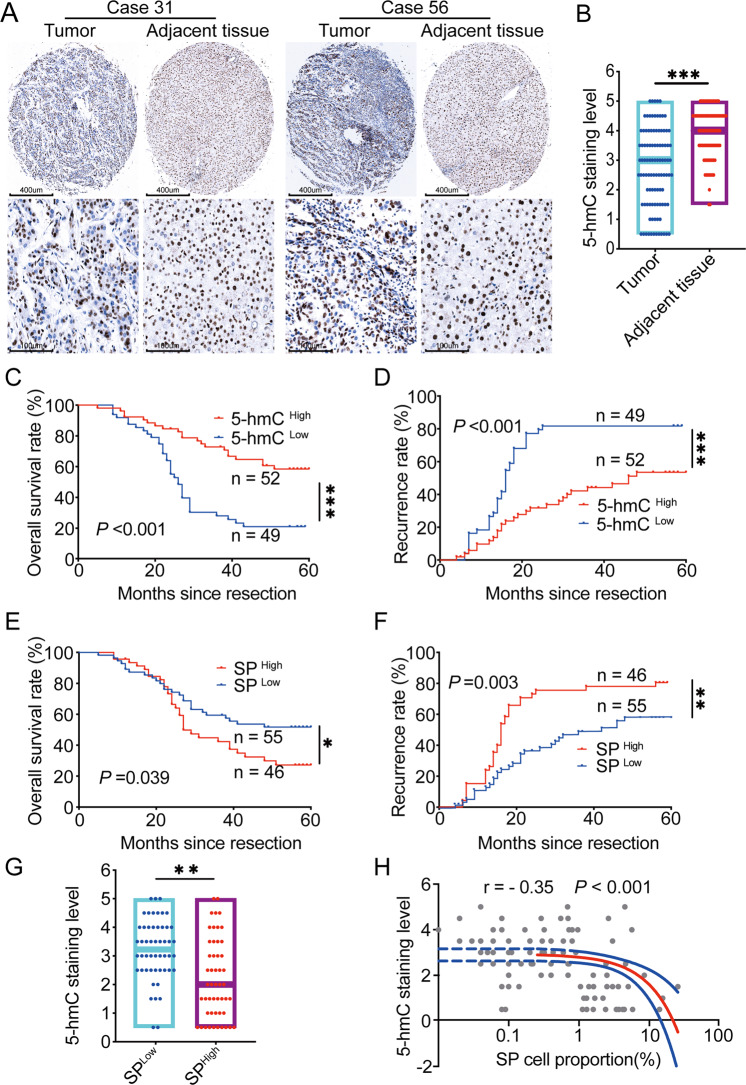
Prognostic significance of 5-hmC level and the proportion of SP cells in HCC. **A** Representative IHC staining images of differential 5-hmC levels in 101 HCC and adjacent nontumor tissues. **B** The staining level of 5-hmC in HCC tumor tissue was significantly weaker than paired adjacent normal tissue; Wilcoxon matched-pairs signed-rank test, ***P < 0.001. **C** Kaplan–Meier estimate of overall survival rate of 101 HCC patients with different levels of 5-hmC; Log-rank test, ***P < 0.001. **D** Kaplan–Meier estimate of cumulative recurrence rate of 101 HCC patients with different levels of 5-hmC; Log-rank test, ***P < 0.001. **E** Kaplan–Meier estimate of the overall survival rate of 101 HCC patients with different proportions of SP cells; Log-rank test, *P* = 0.039. **F** Kaplan–Meier estimate of cumulative recurrence rate of 101 HCC patients with different proportions of SP cells; Log-rank test, *P* = 0.003. **G** The staining level of 5-hmC in SP ^high^ groups was significantly weaker than in SP ^low^; Wilcoxon rank-sum test, *P* = 0.003. **H** Estimation of correlation between 5-hmC staining level and the proportion of SP cells in tumor tissues; Spearman rank correlation, *r* = −0.35, *P* < 0.001.

Our previous studies reported that loss of 5-hmC is an epigenetic hallmark of melanoma [30] and the level of 5-hmC in the genome plays a vital role in tumor growth and stemness [11]. In the present study, we investigated the 5-hmC level in 101 HCC tissues and matched adjacent liver tissues by IHC. Positive staining for 5-hmC was primarily located in the nuclei of liver and tumor cells. The level of 5-hmC in tumor tissues was significantly lower than that in adjacent liver tissues (Fig. 1A, B). Then, with the median of 5-hmC IHC staining score, we divided the whole cohort (101 HCC patients) into 5-hmC^High^ (52 HCC patients with 5-hmC IHC staining score ≥2.5) and 5-hmC^Low^ (49 HCC patients with 5-hmC IHC staining score <2.5) subgroups. As shown in Table 1, a lower level of 5-hmC in the tumor correlated with malignant phenotypes, such as microvascular invasion and poor differentiation. Further, 5-hmC^Low^ patients had a significantly shorter OS (overall survival time, *P* < 0.001) and PFS (progression-free survival time, *P* < 0.001) than those 5-hmC^High^ (Fig. 1C, D). Considering chemoresistance portends to lead an early death, we speculated that there existed a potential link between 5-hmC reduction and chemoresistance.

**Table 1:** Correlation between 5-hmC and clinicopathological characteristics in 101 HCCs.

Variable	5-hmC		*p* value
	Low (*n* = 49)	High (*n* = 52)	
Age (years)			
≤50	22	24	1.000
>50	27	28	
Sex			
Female	12	22	0.091
Male	37	30	
HBsAg			
Negative	4	6	0.742
Positive	45	46	
HCVAb			
Negative	42	45	1.000
Positive	7	7	
Liver cirrhosis			
No	21	27	0.427
Yes	28	25	
Serum AFP, ng/mL			
≤20	4	10	0.151
>20	45	42	
Serum ALT, U/L			
≤75	24	32	0.233
>75	25	20	
Tumor size (diameter, cm)			
≤5	19	25	0.423
>5	30	27	
Tumor encapsulation			
Absent	22	26	0.691
Present	27	26	
Microvascular invasion			
No	18	33	0.010
Yes	31	19	
Tumor number			
Single	29	34	0.542
Multiple	21	18	
Differentiation			
I/II	19	37	0.001
III/IV	30	15	
BCLC staging			
0/A	33	36	1.000
B/C	16	16	

*HBsAg* hepatitis B surface antigen, *BCLC* Barcelona-Clinic Liver Cancer.

It has been broadly described that SP cells are involved in chemoresistance [22]. We believe that analyzing the 5-hmC level of SP cells is a good starting point to reveal the role of 5-hmC reduction in HCC chemoresistance. In the present study, we separated and cultured the SP cells and Hoechst-stained cells (non-SP cells) from Huh 7 cell line. As presented in Supplementary Fig. 1A–C, pharmacodynamics experiments indicated that Huh7 SP cells were less sensitive to oxaliplatin (*P* < 0.01) and 5-FU (*P* = 0.03) than non-SP cells in vitro; besides, oxaliplatin plus 5-FU treated xenograft tumor of Huh 7 SP cells was significantly more extensive than that of non-SP cells, these results verified the chemo-resistant characteristic of SP cells in HCC.

Further, we detected the proportion of SP cells in the tumor tissue of 101 HCC patients, which ranged from 0 to 25.54%, with a median of 0.5%. Importantly, HCC patients with a higher proportion of SP cells (SP proportion ≥0.5%, SP^High^) had a significantly shorter OS (*P* = 0.039) and PFS (*P* = 0.003) than patients with a lower proportion of SP cells (SP proportion <0.5%, SP^Low^) (Fig. 1E, F). Surprisingly, regarding the relationship between SP proportion and 5-hmC reduction, we found that SP^Low^ patients had a higher level of 5-hmC (*P* = 0.003) (Fig. 1G). Correlation analysis also suggested a negative relationship between the level of 5-hmC and the proportion of SP cells (*P* < 0.001) (Fig. 1H). These results indicated that HCC patients with low levels of 5-hmC have a poor prognosis, and 5-hmC reduction is related to a higher proportion of chemo-resistant SP cells.

### Loss of 5-hmC induced by TET2 reduction results in chemotherapy resistance

**Fig. 2:**
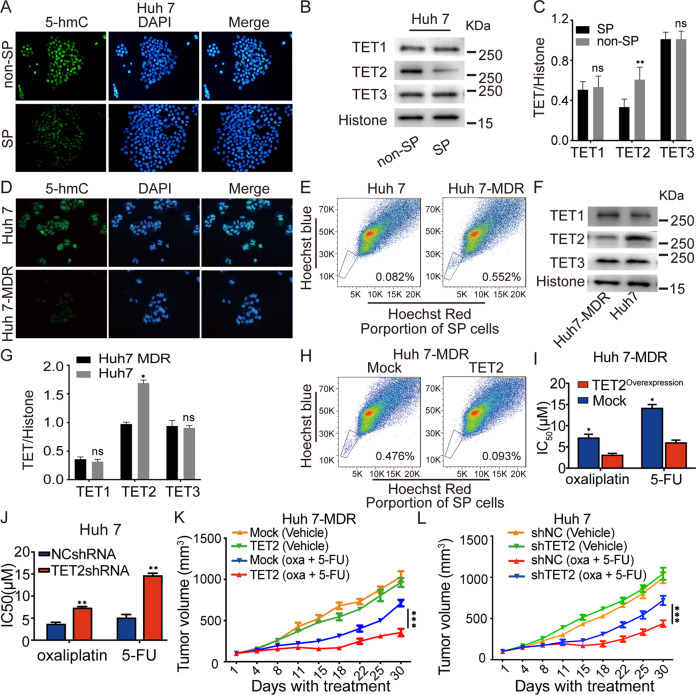
Reduced TET2 expression in SP cells exacerbates chemotherapy resistance in vitro and in vivo. **A** Representative images of 5-hmC in Huh 7 SP and non-SP cells assayed by IF staining. **B**, **C** The expression of TET1, TET2, and TET3 in Huh7 SP and non-SP cells were analyzed by Western blotting; *n* = 3; *t* test, **P < 0.01. **D** Representative images of 5-hmC in Huh 7 and Huh 7-MDR cells assayed by IF staining. **E** The proportion of SP cells in Huh 7 and Huh 7-MDR cells was determined by FCM. **F**, **G** Western blotting analysis was used to detect the expression of TET1-3 in Huh 7 and Huh 7-MDR cells; *n* = 3; *t* test, *P < 0.05. **H** The proportion of SP cells in Huh 7-MDR cells with forced expression of TET2 was assessed by FCM. **I** The chemotherapy sensitivity of Huh 7-MDR cells with forced expression of TET2 assessed by CCK-8 assay in vitro; *n* = 3; *t* test, *P < 0.05. **J** The sensitivity of chemotherapy in Huh 7 cells with reduced expression of TET2 was assessed by CCK-8 assay in vitro; *n* = 3; *t* test, **P < 0.01. **K**, **L** Tumor growth in HCC cells with forced or reduced expression of TET2 was investigated by SC xenograft tumor models against oxaliplatin and 5-FU treatment; *n* = 6; One-way ANOVA, ***P < 0.001. All data are presented as the mean ± SD, *P < 0.05; **P < 0.01; ***P < 0.001.

To further explore the relationship between 5-hmC level and SP cells in HCC, we conducted an Immunofluorescence assay (IF) in vitro and found that Huh7 SP cells have an apparent reduction of 5-hmC level than non-SP cells (Fig. 2A), which is related to TET2 repression rather than TET1 or TET3 deletion (Fig. 2B, C), indicating that TET2 deficiency-induced 5-hmC reduction is relevant to chemoresistance in HCC. To further reveal the underlying mechanism of chemoresistance in HCC, we successfully constructed a multiple-resistant Huh 7 cell line (Huh7-MDR), which is resistant to oxaliplatin and 5-FU. Interestingly, IF and Dot immunoblot assays showed that Huh 7-MDR cells exhibited a significantly lower level of 5-hmC than Huh 7 cells (Fig. 2D and Supplementary Fig. 1D), further verified 5-hmC reduction is involved in chemoresistance; moreover, by conducting flow cytometry, we also found that the proportion of SP cells among Huh 7-MDR cells was significantly elevated than that in Huh 7 cells (Fig. 2E). When compared with Huh 7 cells, the expression of TET2 was decreased in Huh7-MDR cells as well (Fig. 2F, G); further, IF analysis showed that exogenous TET2 overexpression in Huh 7-MDR cells (Huh 7-MDR-TET2) could reverse 5-hmC level (Supplementary Fig. 1E–G); moreover, elevated TET2 expression in Huh7 MDR cells significantly reduced the proportion of SP cells compared to that in Huh 7-MDR-Mock cells (Fig. 2H). Then, we further assessed the chemosensitivity of Huh 7-MDR-Mock and Huh 7-MDR-TET2 cells, and as presented in Fig. 2I, exogenous TET2 overexpression in Huh 7-MDR cells re-sensitized HCC cells to 5-FU and oxaliplatin.

Based on this, we designed three shRNA to knockdown the expression of TET2 in Huh7 cells. As presented in Supplementary Fig. 2A–C, TET2-shRNA1 significantly decreased the expression of TET2 in Huh7 cells, which induced an apparent reduction of the 5-hmC level as well (Supplementary Fig. 2D). Pharmacological experiments also indicated that TET2 deletion significantly affects the sensitivity of Huh7 cells to 5-FU and oxaliplatin (Fig. 2J). Subcutaneous injection of Huh 7-MDR-TET2, Huh 7-MDR-Mock, Huh 7-shTET2, and Huh 7-shNC in mice revealed that when treated with chemotherapy agents, tumor growth of HCC cells with high expression of TET2 was significantly inhibited (Fig. 2K, L and Supplementary Fig. 2E, F). These data suggested that the sensitivity of HCC to chemotherapy agents largely depends on the expression of TET2 and 5-hmC levels.

### TET2-knockdown induced the inhibition of PCAF expression in HCC

**Fig. 3:**
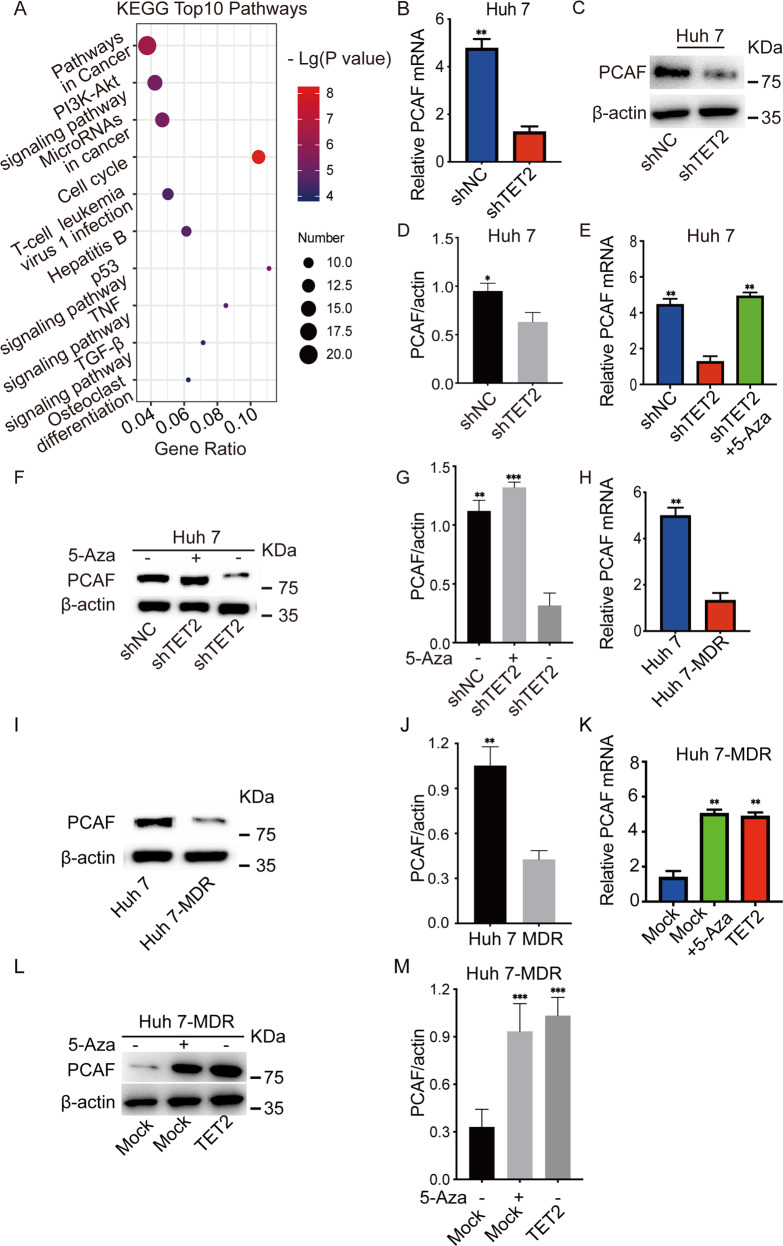
TET2 regulates PCAF expression in HCC cells. **A** Top 10 signaling pathways between Huh7-shTET2 and Huh7-shNC cells determined by KEGG analysis. **B** PCAF mRNA expression in HCC Huh7-shNC and Huh7-shTET2 cells was determined by qRT-PCR analysis; *n* = 3; *t* test, **P < 0.01. **C**, **D** Western blotting depicts the expression of PCAF in HCC Huh7-shNC and Huh7-shTET2 cells; *n* = 3; *t* test, **P < 0.01. **E** PCAF mRNA expression in HCC Huh7-shTET2 cells treated with 5-Aza; *n* = 3; *t* test, **P < 0.01. **F**, **G** The PCAF protein expression in HCC Huh 7 shTET2 cell lines treated with 5-Aza; *n* = 3; *t* test, **P < 0.01; ***P < 0.001. **H**–**J** qRT-PCR analysis and Western blotting showed the PCAF expression in HCC Huh 7 and Huh 7-MDR cells; *n* = 3; t test, **P < 0.01. **K** qRT-PCR analysis of PCAF mRNA expression in Huh 7-MDR-TET2 cells and Huh 7-MDR treated with 5-aza; *n* = 3; t test, **P < 0.01. **L**, **M** Western blotting of PCAF protein expression in Huh 7-MDR-TET2 cells and Huh 7-MDR treated with 5-aza; *n* = 3; t test, ***P < 0.001. All data are presented as the mean ± SD, *P < 0.05; **P < 0.01; ***P < 0.001.

Although plenty of research has indicated that 5-hmC reduction is involved in many malignant processes in the tumor, the specific role of 5-hmC in chemoresistance is still unclear. In the present study, the mRNA-seq analysis showed that 180 genes were downregulated and 329 genes were up-regulated in Huh 7-shTET2 cells compared to Huh 7-shNC cells (Supplementary Table 4). Kyoto Encyclopedia of Genes and Genomes (KEGG) analysis showed that these differentially expressed genes were majorly involved in pathways in cancer, PI3K-AKT signaling, microRNAs in cancer, cell cycle, P53 signaling, TNF signaling, and TGF-β (Fig. 3A). One downregulated gene was histone acetyltransferase PCAF, which promotes granulocytic differentiation in leukemia cells, inhibits self-renewal of breast cancer, and inducts primary hematopoietic cell proliferation and chemotherapy resistance [31–34]. Based on these findings, we hypothesized that reduced PCAF expression is vital for maintaining CSC characteristics, including chemotherapy resistance.

RT-qPCR assay and western blotting revealed that the expression of PCAF in Huh 7 cells was decreased after the knockdown of TET2 (Fig. 3B–D). When these cells were treated with the DNA methyltransferase inhibitor 5-azacytidine (5-Aza) (10 μM) for 48 h, PCAF expression was significantly up-regulated (Fig. 3E–G). Interestingly, PCAF expression was significantly decreased in Huh 7-MDR cells compared to Huh 7 cells (Fig. 3H–J). When Huh 7-MDR cells were transfected with exogenous TET2 or treated with 5-Aza (10 μM), the expression of PCAF was significantly increased at both the mRNA and protein levels (Fig. 3K–M). The above data indicate that TET2-knockdown inhibits PCAF expression by methylation-mediated gene silencing.

### Chemotherapy resistance induced by TET2 deletion depends on the inhibition of PCAF

**Fig. 4:**
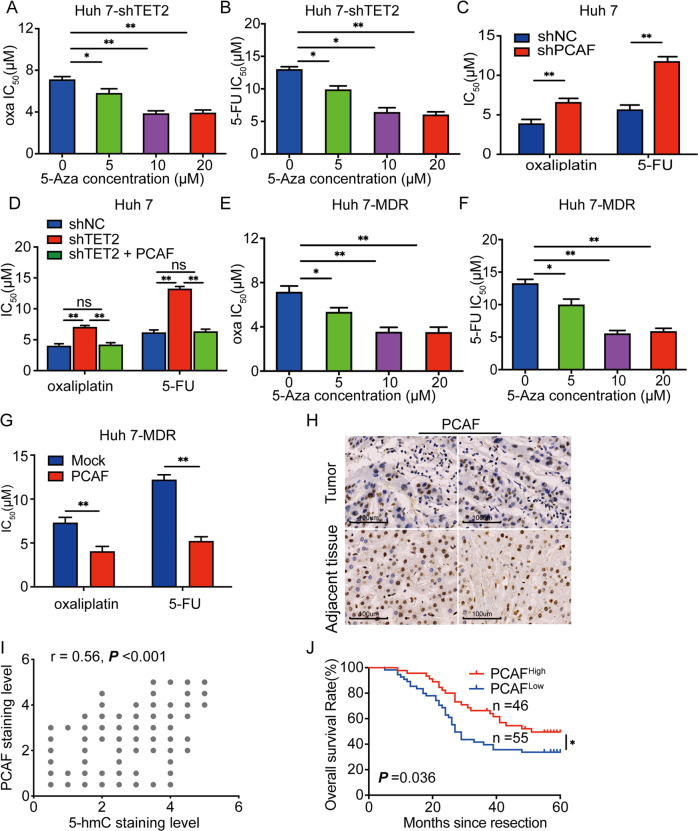
Chemotherapy resistance induced by TET2-knockdown depends on PCAF inhibition. **A** CCK-8 assay showing IC50 of oxaliplatin in Huh 7-shTET2 cells treated with different concentrations of 5-aza; *n* = 3, One-way ANOVA, *P < 0.05; **P < 0.01. **B** CCK-8 assay showing 5-FU IC50 in Huh 7-shTET2 treated with different concentrations of 5-aza; *n* = 3, One-way ANOVA, *P < 0.05; **P < 0.01. **C** CCK-8 assay showing chemotherapy sensitivity in Huh 7 cells with knockdown of PCAF; *n* = 3; *t* test, **P < 0.01. **D** CCK-8 assay showing chemotherapy sensitivity in Huh 7-shTET2 cells with forced expression of PCAF; *n* = 3; One-way ANOVA, **P < 0.01. **E** CCK-8 assay showing oxaliplatin IC50 in Huh 7-MDR cells treated with different concentrations of 5-aza; *n* = 3; One-way ANOVA, *P < 0.05; **P < 0.01. **F** CCK-8 assay showing 5-FU IC50 in Huh 7-MDR cells treated with different concentrations of 5-aza; *n* = 3; One-way ANOVA, *P < 0.05; **P < 0.01. **G** CCK-8 assay to assess the chemotherapy sensitivity of Huh 7-MDR cells with forced expression of PCAF in vitro; *n* = 3; *t* test, **P < 0.01. **H** Representative immunostaining images of PCAF in tumor tissue and adjacent liver tissue in HCC patients. **I** A positive correlation between the level of 5-hmC and PCAF expression was observed in HCC tissues; Spearman rank correlation, r = 0.56; *P* < 0.001. **J** Overall survival analysis of PCAF expression in 101 HCC patients; Log-rank test, *P* = 0.036. All data are presented as mean ± SD; *n* = 3. *P < 0.05; **P < 0.01.

Further, we investigated the role of PCAF in TET2 deficiency-induced chemotherapy resistance in HCC. As presented in Fig. 4A, B, the sensitivity of Huh7-shTET2 cells to oxaliplatin and 5-FU was restored with 5-Aza treatment, verifying that epigenetic modification influences the sensitivity of HCC cells to chemotherapy. By conducting MeDIP/hMeDIP‐qPCR analysis, we found that the 5-hmC level of PCAF was decreased in Huh 7-MDR cells compared to Huh 7 cells. In contrast, the 5-mC level of PCAF was significantly increased in Huh7-MDR cells, which indicates that epigenetic modification of PCAF was related to the chemotherapy resistance in HCC (Supplementary Fig. 3A, B). However, 5-Aza treatment cannot restore TET2 expression in Huh7 MDR cells (Supplementary Fig. 3C–E), suggesting that different from PCAF, TET2 deletion in chemo-resistant HCC cells was not induced by methylation; more complicated mechanisms may be involved in this process. As presented in Supplementary Fig. 3F, we successfully constructed three shRNA for knocking down the expression of PCAF in the Huh7 cells, and PCAF-shRNA1 induced a significant decrease of PCAF in Huh7; further, we constructed a stable Huh7 cell line with knockdown of PCAF using PCAF-shRNA1(named as Huh7-shPCAF cells), and by conducting western blotting, we further confirmed the efficacy of PCAF-shRNA1 in protein levels (Supplementary Fig. 3G, H); moreover, we also constructed stable cell lines with forced expression of PCAF in Huh7 MDR cells and Huh7-shTET2 cells (Supplementary Fig. 3I–L). Pharmacological experiments indicated that interference with PCAF expression in Huh 7 HCC cells decreased the sensitivity to 5-FU and oxaliplatin (Fig. 4C); however, overexpression of PCAF attenuated the resistance to chemotherapy induced by TET2 knockdown (Fig. 4D). 5-Aza also enhanced the sensitivity of Huh 7-MDR cells to oxaliplatin and 5-FU (Fig. 4E, F). Similarly, forced expression of PCAF improved the sensitivity of Huh 7-MDR cells to oxaliplatin and 5-FU (Fig. 4G).

Furthermore, we explored the expression of PCAF in 101 HCC patient tumor tissues and matched adjacent normal tissues via IHC (Fig. 4H). A scatter plot revealed a positive correlation between PCAF protein and 5-hmC levels (Fig. 4I). HCC patients with low PCAF expression had significantly worse prognoses than those with high PCAF expression (Fig. 4J). These data indicate that low levels of 5-hmC induced by TET2 deletion result in chemotherapy resistance in HCC cells in a PCAF-dependent manner.

### Knockdown of TET2 expression activates AKT signaling

**Fig. 5:**
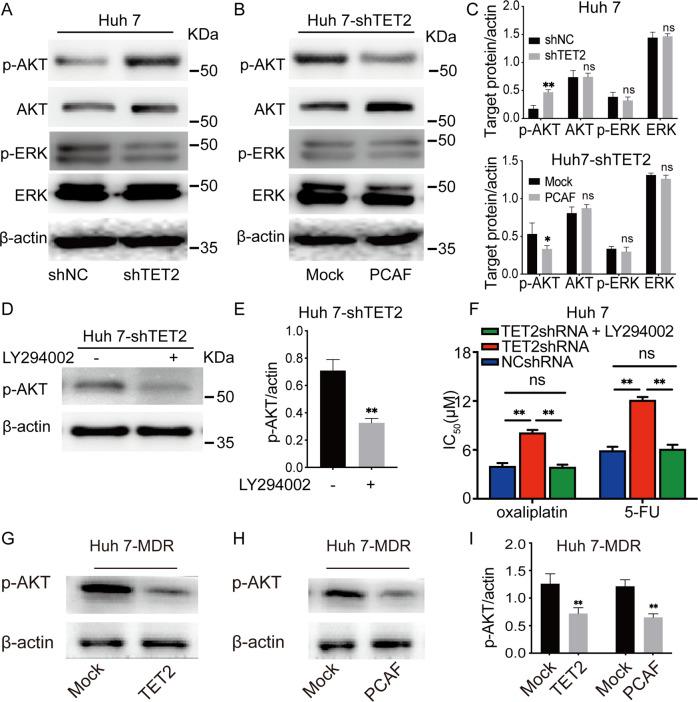
TET2 regulates the PI3K/AKT pathway via PCAF regulation in HCC cells. **A** Western blot showing the expression of AKT, p-AKT, ERK, and p-ERK in HCC Huh 7-shNC and Huh 7-shTET2 cells. **B** Western blot showing the expression of AKT, and ERK in Huh 7-shTET2 cells with forced expression of PCAF. **C** Quantification of AKT, p-AKT, ERK, and p-ERK expression in Huh7, Huh7-shTET2, and PCAF forced Huh7-shTET2 cells; *n* = 3, *t* test, *P < 0.05; **P < 0.01. **D**, **E** Western blot showing the expression of p-AKT in HCC Huh 7-shTET2 cells treated with LY294002; *n* = 3, *t* test, **P < 0.01. **F** CCK-8 assay showing Huh 7-shTET2 sensitivity to oxaliplatin and 5-FU treated with LY294002; *n* = 3, One-way ANOVA, **P < 0. 01. **G**–**I** Western blot showing the expression of p-AKT in Huh 7-MDR cells with forced expression of TET2 or PCAF; *n* = 3, *t* test, **P < 0.01. All data are presented as mean ± SD; *n* = 3, *P < 0.05; **P < 0.01.

Hyperactivation of AKT signaling plays an essential role in the resistance of HCC to chemotherapy agents, such as 5-FU and oxaliplatin [28, 29]. Given increased PCAF expression could inhibit AKT signaling in HCC cells [26, 27], we further scrutinized the results of KEGG analysis with the transcriptome profiles of HCC cells (Fig. 3A), in which pathways in cancer, especially the PI3K-Akt signaling pathway, were significantly hyperactivated. As presented in Fig. 5A, C, unlike other signaling pathways involved in cancer progression, such as ERK signaling, p-AKT was up-regulated considerably in Huh 7 cells after TET2 knockdown. Interestingly, overexpression of PCAF in Huh 7-shTET2 cells decreased p-AKT levels in vitro (Fig. 5B, C), indicating that inhibition of TET2 expression activates AKT signaling, which could be suppressed by overexpression of PCAF in HCC cells. As expected, although LY294002 (an inhibitor of PI3K) treatment did not affect the expression of AKT or PCAF (Supplementary Fig. 4A, B), p-AKT expression in Huh 7-shTET2 cells was significantly decreased (Fig. 5D, E). Consistently, Huh 7-shTET2 cells treated with LY294002 rescued the sensitivity of HCC cells to chemotherapy agents (Fig. 5F). Furthermore, p-AKT expression was inhibited in Huh 7-MDR cells after overexpression of TET2, while PCAF expression was up-regulated (Fig. 5G, I and Supplementary Fig. 4C, D). Similarly, p-AKT expression was reduced in Huh 7-MDR cells after overexpression of PCAF (Fig. 5H, I and Supplementary Fig. 4E, F). Together, these data indicate that hyperactivation of AKT signaling is likely involved in chemotherapy resistance induced by TET2/PCAF reduction.

### Knockdown of TET2 inhibited apoptosis and promoted colony formation in HCC cells

**Fig. 6:**
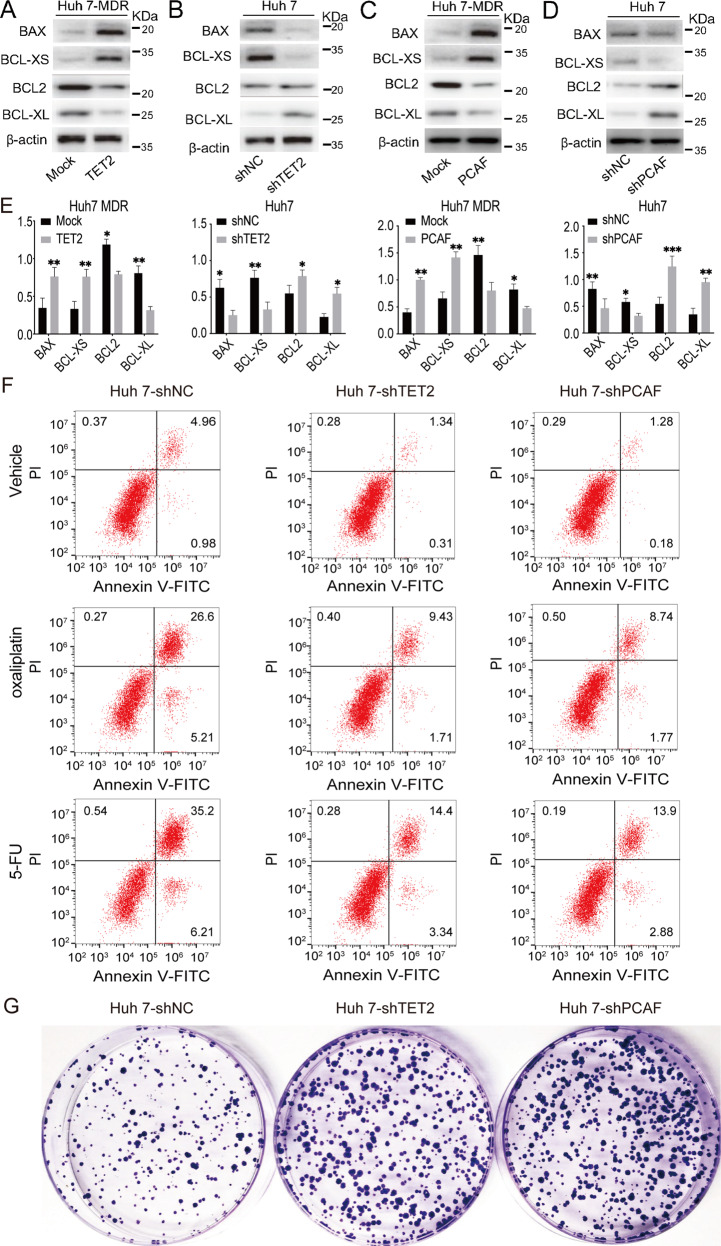
TET2 induces HCC apoptosis through a TET2/PCAF axis. **A** Western blot showing the apoptosis-related and anti-apoptosis proteins expressed in Huh 7-MDR cells with forced expression of TET2. **B** Western blot showing the apoptosis-related and anti-apoptosis proteins expressed in Huh 7 cells with reduced expression of TET2. **C** Western blot showing apoptosis-related and anti-apoptosis protein expression in Huh 7-MDR cells with forced expression of PCAF. **D** Western blot showing the apoptosis-related and anti-apoptosis protein expression in Huh 7 cells with reduced expression of PCAF. **E** Quantification of apoptosis-related protein expression in Huh7, Huh7-shPCAF, Huh7-shTET2, Huh7 MDR and Huh7 MDR with forced expression of TET2 or PCAF; *n* = 3, *t* test, *P < 0.05; **P < 0.01; ***P < 0.001. **F** The apoptosis rate of oxaliplatin or 5-FU treated Huh7-NC, Huh7-shTET2, and Huh7-shPCAF cells were assessed by FCM. **G** Proliferation in HCC cells with reduced expression of TET2 or PCAF was assessed by clonal formation assay.

A previous study has indicated that PCAF prompts cell apoptosis and AKT signaling inactivation in HCC [26]. Here, we investigated whether AKT signaling activation-induced survival is related to chemoresistance. As shown in Fig. 6A, E, overexpression of TET2 in Huh 7-MDR cells resulted in reduced expression of the antiapoptotic proteins BCL2 and BCL-XL, and the apoptotic proteins BAX and BCL-XS were increased. In contrast, the expression of antiapoptotic proteins was significantly up-regulated, and apoptotic proteins were significantly downregulated in Huh 7 cells after TET2 knockdown (Fig. 6B, E). Interestingly, the conversion of apoptosis-related protein expression in Huh 7-MDR-PCAF was along with Huh 7-MDR-TET2 cells (Fig. 6C, E). Correspondingly, the changes in apoptosis-related protein in Huh 7-shPCAF cells were the same as those in Huh 7-shTET2 cells (Fig. 6D, E). Apoptosis assays showed that reduced expression of TET2 or PCAF significantly suppressed apoptosis in Huh 7 cells when treated with oxaliplatin (4uM, IC50 toward Huh7 cells) or 5-FU (6uM, IC50 toward Huh7 cells) for 48 h (Fig. 6F); in contrast, forced expression of TET2 and PCAF substantially prompted apoptosis in Huh7-MDR cells (Supplementary Fig. 4G); moreover, overexpression of TET2 and PCAF inhibited colony formation in Huh 7-MDR cells; in contrast, inhibition of TET2 and PCAF expression promoted colony formation in Huh 7 cells (Fig. 6G and Supplementary Fig. 4H).

### TET2 deficiency caused 5-hmC reduction leads to chemotherapy resistance in HCC patients

**Fig. 7:**
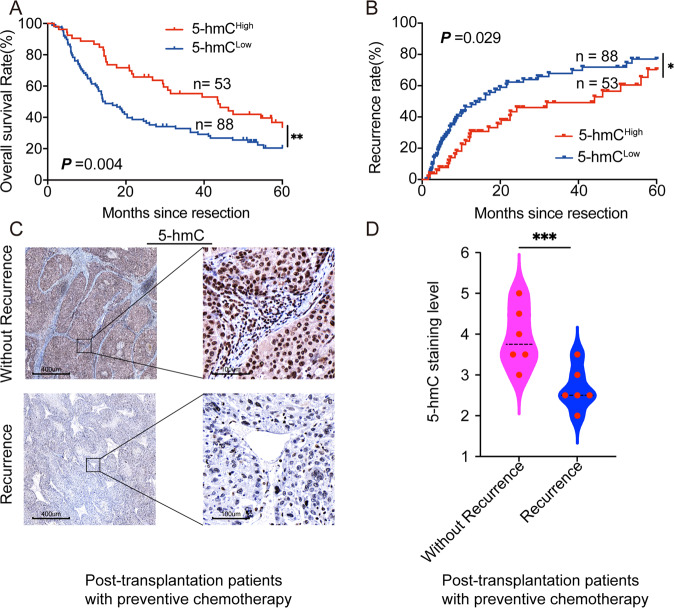
Loss of 5-hmC contributes to the resistance against oxaliplatin and 5-FU. **A**, **B** Kaplan–Meier estimates overall survival and cumulative recurrence rate of advanced recurrent HCC patients after TACE combined oxaliplatin and 5-FU treatment with different levels of 5-mC; Log-rank test, *P < 0.05; **P < 0.01. **C**, **D** 5-hmC staining in post-transplantation, preventive chemotherapy-treated HCC patients with or without recurrence; Wilcoxon rank-sum test, ***P < 0.01. All data are presented as mean ± SD; *n* = 3. **P < 0.001; ***P < 0.01.

Retrospective data were analyzed from 141 patients with advanced, recurrent HCC who underwent TACE administered with oxaliplatin and 5-FU from 2 to 60 months after liver resection. The 5-hmC level was measured in HCC tissues from the above 141 patients, and Kaplan–Meier survival analysis showed that the OS rate of HCC patients with high levels of 5-hmC was much higher than that of patients with low levels of 5-hmC. The median OS was 14.4 months in the 5-hmC^Low^ group and 43.6 months in the 5-hmC^High^ group (hazard ratio: 1.780; 95% confidence interval: 1.198–2.644; Log-rank test, *P* = 0.004) (Fig. 7A). Further, we found that patients with low 5-hmC levels had a significantly higher cumulative recurrence rate than those with high 5-hmC levels (hazard ratio: 1.629; 95% confidence interval: 1.049–2.529; Log-rank test, *P* = 0.029) (Fig. 7B); moreover, a retrospective study was analyzed with 12 HCC patients who received preventive chemotherapy with oxaliplatin and 5-FU following liver transplantation between 2017 and 2020. Six of twelve patients were confirmed with recurrence during chemotherapy, suggesting that these patients existed resistance to oxaliplatin and 5-FU. Then we conducted IHC in the tumor samples of these patients; as presented in Fig. 7C, D, the level of 5-hmC significantly receded in tumors of patients with recurrence during chemotherapy (*P* < 0.01). These results indicate that reduced TET2 leads to DNA methylation imbalance and induces chemoresistance in HCC patients.

## Discussion

Chemotherapy resistance limits long-term survival in patients with malignant tumors [35]. Abnormal 5-mC modification is a prominent epigenetic feature of cancer. Growing evidence has established a link between abnormal DNA methylation and chemotherapy resistance [36–38]. Although the loss of 5-hmC results in poor prognoses among HCC patients, the mechanism of 5-hmC reduction-induced chemotherapy resistance in HCC remains to be established. In the present study, we show that 5-hmC levels are significantly reduced in HCC tissues, and 5-hmC reduction is a valuable factor for prognostic prediction in HCC; besides, patients with a low level of 5-hmC hardly benefit from adjuvant TACE administered with oxaliplatin and 5-FU treatment. These findings support that the global reduction of 5-hmC in HCC plays an essential role in chemotherapy resistance and tumor progression.

The TET family converts 5-mC to 5-hmC and thus regulates gene transcription. TET2 mutational inactivation decreases 5-hmC levels in several malignant tumors [39, 40]. SP cells could be separated from many normal organs and malignant tissues, which are preferred to be resistant to various chemotherapy agents through different mechanisms [4, 5]. In the present study, we verified that Huh7 SP cells are less sensitive to chemotherapy agents, and the proportion of SP cells in Huh7 MDR cells is significantly elevated; furthermore, patients with a higher proportion of SP cells have an unfavorable prognosis, which indicates SP cells are involved in chemoresistance and progression of HCC. Interestingly, 5-hmC levels significantly correlate with the proportion of SP cells in tumor tissues; moreover, TET2 expression is reduced substantially in Huh7 SP cells than in non-SP cells, along with the decline of 5-hmC level, and overexpression of TET2 in Huh7 MDR cells increased 5-hmC level and reduced SP proportion. These results suggest that 5-hmC reduction induced by TET2 deficiency may be related to chemoresistance in HCC.

PCAF-induced Akt signaling inactivation plays an essential role in HCC chemoresistance and progression. Here, we determined that the knockdown of TET2 in Huh 7 cells resulted in the downregulation of PCAF. Further, the downregulation of PCAF expression could be rescued by 5-Aza treatment in Huh 7-shTET2 cells. Clinical data also showed a positive relation between PCAF expression and 5-hmC levels in HCC patients. These data suggest that TET2 reduction-induced 5-hmC deficiency inhibits PCAF expression. Interestingly, AKT signaling was activated in Huh 7 cells after the knockdown of TET2, and overexpression of PCAF in Huh 7-shTET2 cells resulted in AKT inactivation; moreover, the knockdown of TET2 resulted in resistance to chemotherapy, which could be attenuated by a PI3K/AKT inhibitor. In Huh 7-MDR cells, both overexpression of TET2 and upregulation of PCAF significantly inhibited AKT signaling. These results indicate that TET2 deficiency induces chemotherapy resistance in HCC cells via the 5-hmC/PCAF/AKT axis.

Further, since apoptosis reduction is one of the critical features of chemotherapy-resistant tumor cells, we examined the expression of apoptosis-related proteins in HCC cells with different levels of TET2/PCAF [41]. Knockdown of TET2/PCAF suppressed apoptosis and promoted cell proliferation in Huh 7 cells, and overexpression of TET2/PCAF in HUH 7-MDR cells resulted in increased apoptosis and decreased cell proliferation. These results indicate that reduced TET2/PCAF suppressed apoptosis and promoted proliferation in HCC.

However, in the absence of chemotherapeutic treatments, in vivo experiments presented that the tumorigenicity of Huh7-MDR-TET2 and Huh7- shTET2 cells failed to reach a statistical significance compared to control cells. We believe such a difference may be derived from the discrepancies in tumor development conditions in vivo and in vitro. Unlike in vitro, the proliferative potential of tumor cells in vivo may be restricted by the shortage of sustainable molecules and the disruption of complicated regulation signals. Here, despite the expression of TET2 in HCC cells didn’t affect tumor growth in vivo when treated with vehicles, the chemo-resistant potential of Huh 7 MDR and TET2 deficient Huh7 cells was preserved, which further indicated that TET2 reduction greatly affected the chemo-resistant phenotype of HCC cells whether in vivo or in vivo.

In the present study, we have revealed that PCAF plays a vital role in chemoresistance through epigenetic modification, and 5-Aza treatment could restore PCAF expression in chemotherapy-resistant Huh7 MDR cells; although it has been reported that TET2 expression could be regulated by methylation as well [42], there is no significant elevation of TET2 in Huh7-MDR cells with disposes of 5-Aza, which suggested that TET2 deletion in chemo-resistant HCC cells was not primarily induced by methylation, more complicated mechanisms may be involved in this process, further investigations should be conducted to reveal the mechanism of TET2 deletion in HCC chemoresistance formation.

In conclusion, the loss of 5-hmC plays a pivotal role in developing chemotherapy resistance and tumor progression in HCC. 5-hmC reduction inhibits the expression of PCAF, activates AKT signaling, and suppresses apoptosis, thus inducing chemotherapy resistance and HCC progression. Our studies not only showed that reducing 5-hmC levels related to poor prognosis but also established the mechanism of chemotherapy resistance in HCC. Based on our findings, the loss of 5-hmC may be a biomarker in predicting the response to chemotherapy agents and a new therapeutic target for chemotherapy-resistant HCC.

## Materials and methods

### Cell lines and transfection experiments

Huh 7 (purchased from the Chinese Academy of Sciences Shanghai Branch Cell Bank, Shanghai, China) and chemotherapy-resistant Huh 7 cells (Huh 7-MDR cells) were used in this study. The method for establishing stable Huh 7-MDR cells was described previously with minor modifications [43]. Briefly, Huh 7 cells were initially incubated in 1 μM 5-fluorouracil (5-FU) and 0.5 μM oxaliplatin for 4 weeks. When stable growth was achieved, the concentrations of 5-FU and oxaliplatin were increased every 4 weeks over 6 months to a maximum dose of 6 μM 5-FU and 3 μM oxaliplatin. Before experimental use, Huh 7-MDR cells were maintained in 5-FU and oxaliplatin-free culture medium and passaged at least three times. These cells were grown in Dulbecco’s modified Eagle medium (DMEM) supplemented with 10% FBS, 100 U/ml penicillin, and 0.1 mg/ml streptomycin.

The pU6-vshRNA-CMV-Puro lentiviral vectors (pU6 is a lentiviral vector) were purchased from Shanghai GeneChem Co. Ltd., and the shRNAs were constructed and synthesized by Shanghai GeneChem Co., Ltd. The lentiviral vectors were transfected into cells according to the manufacturer’s instructions. TET2 and P300/CBP-associated factor (PCAF) cDNAs were cloned into the pLVX vector (Genomeditech, Shanghai, China) and transfected into HCC cells using Lipofectamine 2000 reagent (Invitrogen) according to the manufacturer’s instructions. Stably transfected clones were validated by qRT-PCR and western blotting. The shRNA target sequences are listed in Supplementary Table 1.

### Tumor sample collection, tissue microarrays (TMAs) construction, and immunohistochemistry (IHC)

Two cohorts from the Liver Cancer Institute of Fudan University (Shanghai, China) were enrolled in the present study between 2004 and 2008. One of the cohorts consisted of 101 HCC curative patients who underwent radical resection. The other consisted of 141 advanced, recurrent HCC patients who had undergone liver resection followed by adjuvant TACE combined with oxaliplatin and 5-FU treatment between 2004 and 2007. The specimens from two cohorts were collected and well-preserved. Tumor samples from 12 HCC patients who received liver transplantation and adjuvant chemotherapy with oxaliplatin and 5-FU between 2017 and 2020 were also collected. Paraffin blocks were selected only based on the availability of suitable formalin-fixed, paraffin-embedded tissue and complete clinicopathologic and follow-up data for the patients. The histopathological diagnosis was based on the World Health Organization criteria. Ethical approval was obtained from the Zhongshan Hospital Research Ethics Committee and written informed consent was obtained from each patient.

TMA construction and IHC were performed as previously described in our study [44], and the level of 5-hmC and the expression of PCAF were detected by IHC. Tissues from HCC patients were reviewed histologically by hematoxylin and eosin (H&E) staining, and representative areas were premarked in paraffin blocks away from necrotic and hemorrhagic materials. Sections (4-μm thick) were placed on slides coated with (3-aminopropyl) triethoxysilane (APTES). 5-hmC level and PCAF expression were evaluated by an integrated imaging system (MetaMorph Imaging System version 3.0; Universal Imaging Corp, Buckinghamshire, UK). The antibodies are listed in Supplementary Table 2.

### Real-time quantitative reverse transcription PCR (RT-qPCR) and RNA sequencing

RNA extraction, RT-qPCR analysis, and sequencing were performed with three technical replicates as previously described [45–47]. Briefly, total RNA was extracted by Trizol (Invitrogen) from Huh 7 and modified Huh7 cells; the quantity and quality of extracted total RNA were determined by Agilent 2100 Bioanalyzer (Agilent). Then, the isolated RNA was reverse transcribed into cDNA with Superscript III first strand synthesis kit (Invitrogen) according to users‘ instructions. RT-qPCR was performed on ABI 7500 real-time cycler (Thermo Fisher Scientific) with 40 amplification cycles of PCR. Related primer sequences for PCR are listed in Supplementary Table 3.

mRNA samples were sequenced by Illumina HiSeq X10 platform (Illumina) according to the manufacturer’s protocol; then, the reads from fastq documents were aligned to the human genomes (build hg19/GRCh37); further, the differentially expressed genes were analyzed by DESeq2 according to standard procedures.

### Chromatin Immunoprecipitation (ChIP)

Genomic DNA was extracted with The PureLink Genomic DNA Mini Kit (K18200, Thermo). Purified Genomic DNA was sonicated to an average size of around 200 bp (range: 100–500 bp) with a bioruptor (Diagenode). DNA fragments were end-repaired, A-tailed, and custom TruSeq adapters (non-methylated) were ligated using the TruSeq DNA sample preparation Kit (Illumina). The DNA fragments were ligated with adapters immunoprecipitated with Protein A + G Magnetic beads coupled with a 5mC antibody or 5hmC antibody. For MeDIP/hMeDIP‐qPCR analysis, we performed real‐time qPCR with input DNA and the immunoprecipitated methylated DNA using SYBR Premix EX Taq (TaKaRa Bio).

### Western blotting, dot blot, and immunofluorescence (IF) assays

Western blotting and IF were performed as previously described [45]. Immunoblot assays were performed as previously described [11]. Briefly, protein samples from Huh7 and modified Huh7 cells were lysed in a Cell Lysis solution (Sigma) containing protease inhibitors and phosphatase inhibitors. Then, the protein samples were heated at 95 °C, for 5 min for denaturation. Equal total protein samples were separated by 7.5%- 12.5% SDS-PAGE and transferred to PVDF membranes. The membrane was blocked by 5% milk in TBS-Tween 20 and then incubated with primary antibody and secondary antibody in turn. Finally, the blots were detected by ECL Western blotting Detection Reagent (Abcam), and the results were analyzed by ImageJ software.

For the IF assay, we fixed the cell samples in 4% paraformaldehyde and permeabilized them by 0.5% Triton X-100. Then, the samples were incubated with primary antibodies and secondary antibodies in turn. Images were collected by microscopy Zeiss LSM700 system. For performing Immunoblot assays, the Purified genomic DNA samples were spotted on a nitrocellulose membrane (Abcam) and baked at 80 °C for over 2 h. Following this, membranes were blocked by 3% BSA and incubated with primary antibodies against 5-hmC or 5-mC. Anti-Rabbit IgG was applied as the secondary antibody, followed by visualization with ECL Reagent(Abcam).

### Cell Counting Kit-8 (CCK-8) assay

The influence of oxaliplatin and 5-FU in proliferation was measured by CCK-8 assay as described in our previous study [48]. In brief, cells were plated in 96-well plates at 2 × 10^3^ cells per well. After 24 h, the cells were plated in a complete medium containing the indicated concentrations of oxaliplatin (Hisunpharm, Taizhou, China), 5-FU (Jinghua, Nantong, China), or vehicle control. Then, the plates were incubated at 37 °C. After 72 h, the CCK-8 (Dojindo, Kumamoto, Japan) solution (10 μL) was added to 100 μL of culture media, and the optical density was measured at 450 nm.

### Colony formation assay

Colony formation was measured as described in our previous study [48]. In brief, HCC cells were seeded into cell culture plates at 1,000 cells/plate. Ten days later, the cells were washed with phosphate-buffered saline (PBS) and fixed with 4% paraformaldehyde. Then, the cells were stained with crystal violet, and the colonies were counted.

### Flow cytometry (FCM)

SP cells were a small fraction of cells that can efflux the fluorescent dye Hoechst 33342 in flow cytometry detection. In the present study, we sorted SP cells and detected their percentage by FCM as described previously [17, 49]. In brief, cells were collected from dishes, washed in PBS, and then suspended in DMEM containing 2% FBS and 10 mmol/L hydroxyethyl piperazine-2-ethane sulfonic acid (HEPES). Cell suspensions at concentrations of 1 × 10^6^ cells/mL were stained with 20 µg/ml Hoechst 33342 (Invitrogen, Carlsbad, CA, USA) either alone or with 25 µg/ml verapamil (Sigma-Aldrich, USA) in a 37 °C-water bath for 90 min (gently shaking at intervals of 10 min). After incubation, the cell suspensions were centrifuged at 4 °C, and the cell pellets were resuspended in precooled Hanks balanced salt solution (HBSS) (Invitrogen) containing 2 μL/mL propidium iodide (PI). SP cell analysis and sorting were performed with an Epics Altra Flow Cell Sorter (Beckman Coulter; Fullerton, CA, USA) with a 488-nm argon laser and an INNOVA 90-CA5 ultraviolet laser (Coherent; Santa Clara, CA, USA). The Hoechst dye was excited by a 351-nm ultraviolet laser, and the fluorescence emission was collected through 450DF20 (Hoechst blue) and 675ALP filters (Hoechst red). Side population (SP) gating was defined as cells that were removed by verapamil treatment, then, the percentages of SP population and non-SP were recorded, and SP cells and non-SP cells were sorted and cultured in DMEM media added with 10% fetal bovine serum (Gbico, 10099-141) and 1% penicillin-streptomycin (Beyotime, C0222) for following experiments.

Apoptosis analysis by flow cytometry was measured as described in our previous study [50]. In brief, apoptotic cells were evaluated using Annexin-V/FITC Apoptosis Detection Kit I (BD Pharmingen, San Jose, Calif) according to the manufacturer’s protocol. The stained cells were then analyzed with a FACS Calibur flow cytometer (BD Biosciences), and the data were analyzed using FlowJo software (TreeStar Inc., Ashland, OR).

### Animal experiment

HCC cells (3 × 10^6^) were subcutaneously injected into 4- to 6-week-old nude mice. When the tumors reached a mean tumor volume of 150 mm^3^, the mice were randomly allocated into two groups (*n* = 6) and assigned to receive one of the following two injections: (i) vehicle (sterile PBS); (ii) 5-FU (25 mg/kg) and oxaliplatin (5 mg/kg) dissolved in sterile saline. Injections were administered intraperitoneally 3 times per week for 2 weeks. Tumor volume was measured every other day in two dimensions with Vernier calipers. Tumor volume was calculated using the formula [length × (width^2^)] × 0.5. Blinding was not involved in the present study. Mice were manipulated and housed according to protocols approved by the Shanghai Medical Experimental Animal Care Commission.

### Evaluation of 5-hmC and PCAF staining

A positive reaction for 5-hmC and PCAF was scored in four grade categories depending on the intensity of the staining (0–3; 0 = absent; 1 = low; 2 = medium; 3 = high), and the percentage of positively stained cells was scored in 5 groups:0 (negative staining), 0.5 (1 to 25%), 1 (26 to 50%), 1.5 (51 to 75%), 2 (76 to 100%). In cases of discrepancies between the duplicated cores, the higher score of the two tissues was taken as the final score. The sum of the intensity and percentage scores was used as the final staining score. Each patient’s expression level of 5-hmC or PCAF was classified as either high (expression score >2.5) or low (expression score ≤2.5). Then, the correlations between the two groups were detected by analysis of Pearson’s correlation coefficient and Spearman’s rank correlation coefficient.

### Statistical analysis

Statistical analysis was performed with SPSS 25.0 software (Chicago, IL), GraphPad Prism 9.0 (San Diego, CA, USA), and R (version 4.0.2, R foundation for statistical, Vienna, Austria). Values are expressed as the mean ± standard deviation (SD). Student’s *t* test, Wilcoxon matched-pairs signed-rank test, and Wilcoxon rank-sum test were used for comparisons between groups. Categorical data were analyzed by the chi-square or Fisher’s exact tests. The correlation between PCAF and 5-hmC was assessed by Spearman correlation analysis. The cumulative recurrence and survival rates were analyzed using the Kaplan–Meier method and the Log-rank test. All tests were two-tailed, and *P* < 0.05 indicated statistical significance.

## Supplementary information

Supplementary Figure Legend

Supplemental file_Uncropped WB

aj-checklist for CDDIS-22-0660RRR

Supplementary Figure 1

Supplementary Figure 2

Supplementary Figure 3

Supplementary Figure 4

Supplementary Table 1

Supplementary Table 2

Supplementary Table 3

Supplementary Table 4

## Data Availability

The data of this study are available from the corresponding authors for reasonable requests.

## Material availability

The materials of this study are available from the corresponding authors for reasonable requests.
