# Supplementary material for: Loss of 5-hydroxymethylcytosine induces chemotherapy resistance in hepatocellular carcinoma via the 5-hmC/PCAF/AKT axis
Source: Cell Death Dis. 2023 Feb 2;14(2):79. doi: 10.1038/s41419-022-05406-3 (PMC9895048; doi:10.1038/s41419-022-05406-3)
Supplement: Supplementary file 1 — Supplementary Figure Legend [file 41419_2022_5406_MOESM1_ESM.docx]

**Supplementary Figure 1 Modified epigenetic features and SP cells related to chemo-resistance in HCC.**

(A) CCK-8 assay showing chemotherapy sensitivity in Huh 7 side population (SP) and non-SP population cells; n = 3; t-test, **P* < 0.05, ***P* < 0.01.

(B) Chemotherapy sensitivity of Huh7 SP and non-SP cells was investigated by xenograft tumour models treated with oxaliplatin and 5-FU; n = 6; t-test, ****P* < 0.001.

(C)Tumors of Huh7 SP cells were less sensitive to oxaliplatin and 5-FU treatment than Huh7 non-SP cells; n = 6.

(D) Dot blot was used to detect the 5-hmC level in HCC Huh 7 cells and Huh 7-MDR cells.

(E & F) Western blotting was used to detect the TET2 expression in HCC Huh 7-MDR cells with forced expression of TET2; n=3; t-test, **P* < 0.05.

(G) Representative images of 5-hmC in Huh 7-MDR cells with forced expression of TET2 assessed by IF staining.

The data are presented as the mean ± SD; **P* < 0.05, ***P* < 0.01, ****P* < 0.001.

**Supplementary Figure 2 TET2 expression related to chemo-resistance in HCC cells**

(A) RT-qPCR analysis was used to detect the TET2 mRNA expression in HCC Huh 7 cells transfected with TET2shRNA; n = 3; t-test, **P* < 0.05, ***P* < 0.01.

(B&C) Western blotting analysis was used to detect the TET2 protein expression in HCC Huh 7 cells transfected with TET2shRNA; n = 3; t-test, **P* < 0.05.

(D) 5-hmC level in Huh 7 cells with reduced expression of TET2 was assessed by an IF assay.

(E&F) Tumours of HCC cells with low expression of TET2 were less sensitive to oxaliplatin and 5-FU treatment than TET2 highly expressed ones; n=6.

The data are presented as the mean ± SD; **P* < 0.05, ***P* < 0.01, ****P* < 0.001.

**Supplementary Figure 3 Methylation and modification of PCAF in chemo-resistant HCC cells.**

(A&B) 5-hmC and 5-mC level of PCAF gene were examined by hMeDIP/MeDIP‐qPCR in Huh 7 and Huh 7-MDR cells; n = 3; t-test, **P < 0.01, ***P < 0.01.

(C) TET2 mRNA expression in HCC Huh 7, Huh7 MDR, and Huh7 MDR treated with 5-Aza; n = 3; t-test, ***P* < 0.01.

(D&E) The expression of TET2 in HCC Huh 7, Huh7 MDR, and Huh7 MDR treated with 5-Aza was detected by Western blotting; n = 3; t-test, ***P* < 0.01.

(F) RT-qPCR analysis was used to detect the PCAF mRNA expression in HCC Huh 7 cells transfected with PCAFshRNA1-3; n = 3; t-test, **P*< 0.05, ***P* < 0.01.

(G&H) Western blotting analysis was used to detect the PCAF protein expression in HCC Huh 7 cells transfected with PCAFshRNA; n = 3; t-test, **P*< 0.05.

(I) Western blotting analysis was used to detect the PCAF protein expression in HCC Huh 7-shTET2 cells transfected with PCAF cDNA.

(J) Quantification of PCAF expression in Huh7-shTET2 cells transfected with PCAF cDNA; n=3; t-test, ***P* < 0.01.

(K&L) Western blotting analysis was used to detect the PCAF protein expression in Huh 7-MDR cells transfected with PCAF cDNA; n=3; t-test, ***P* < 0.01

All data are presented as the mean ± SD; n = 3; **P* < 0.05, ***P* < 0.01, ****P* < 0.001.

**Supplementary Figure 4 TET2 induces HCC apoptosis through a PCAF-related pathway.**

(A&B) Western blot showing the expression of AKT and PCAF in HCC Huh 7-shTET2 cells treated with LY294002; n = 3; t-test, ns: not significant.

(C-F) Western blot showing the expression of AKT and PCAF in Huh 7-MDR cells with forced expression of TET2 or PCAF; n = 3; t-test, ***P* < 0.01, ns: not significant.

(G) The apoptosis rate of Huh 7-MDR cells with forced expression of TET2 or PCAF was assessed by FCM.

(H) Proliferation in Huh 7-MDR cells with forced expression of TET2 or PCAF was assessed by clonal formation assay.
