# Supplementary material for: Loss of 5-hydroxymethylcytosine induces chemotherapy resistance in hepatocellular carcinoma via the 5-hmC/PCAF/AKT axis
Source: Cell Death Dis. 2023 Feb 2;14(2):79. doi: 10.1038/s41419-022-05406-3 (PMC9895048; doi:10.1038/s41419-022-05406-3)

Full and uncropped western blot for Figure2B
Lanes 1,2 are on the Figure

TET1
Histone

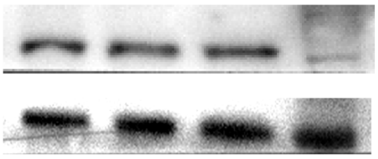

1 2
non-SP SP

TET2
Histone

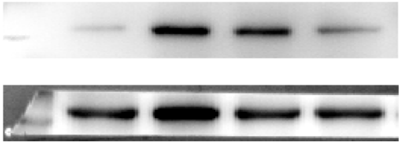

1 2
non-SP SP

TET3
Histone

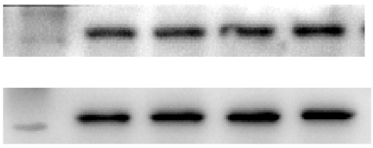

1 2
non-SP SP

Full and uncropped western blot for Figure2F
Lanes 1,2 are on the Figure

TET1
Histone

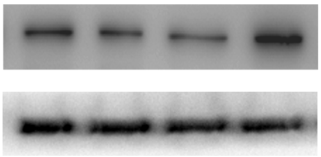

1 2
MDR Huh7

TET1
Histone

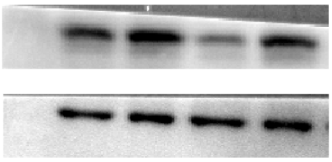

1 2
MDR Huh7

TET3
Histone

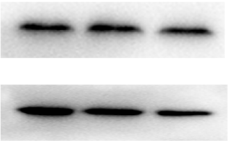

1 2
MDR Huh7

Full and uncropped western blot for Figure3C
Lanes 1,2 are on the Figure

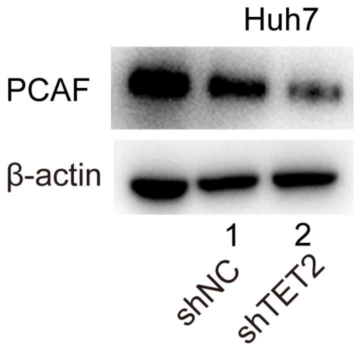

Full and uncropped western blot for Figure3F&I

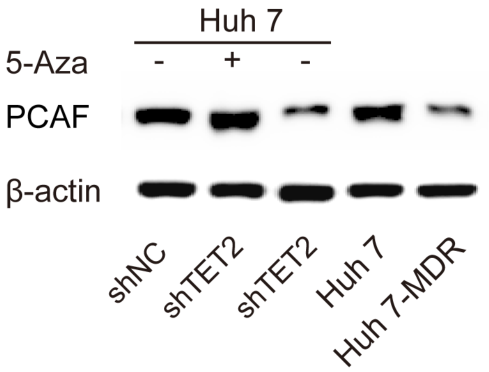

Full and uncropped western blot for Figure3L

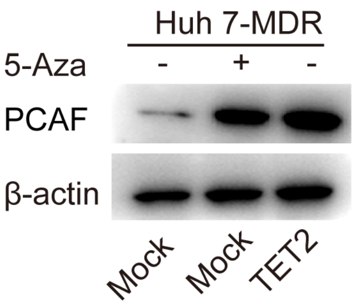

Full and uncropped western blot for Figure5A&B

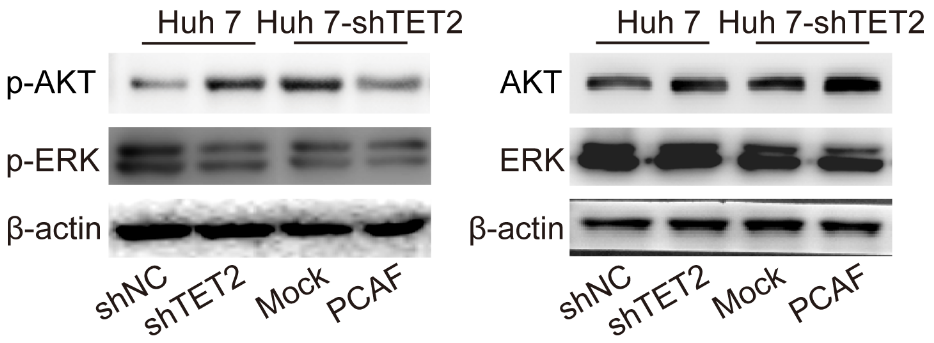

Full and uncropped western blot for Figure5D

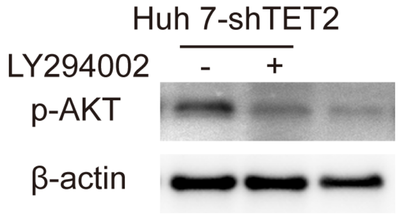

Full and uncropped western blot for Figure5G&H

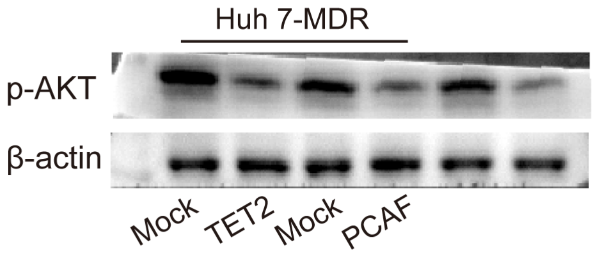

Full and uncropped western blot for Figure6A-D

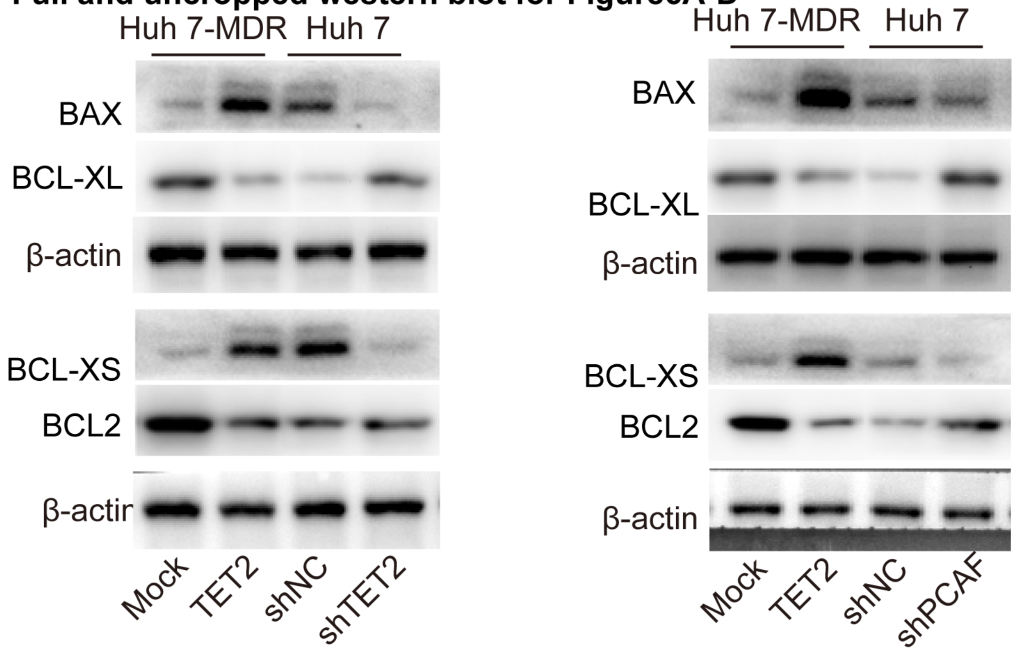

Full and uncropped western blot for Figure S1E&S2B

Lanes 1,2 are on the Figure

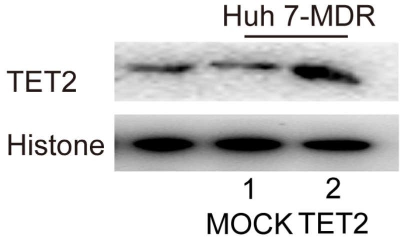

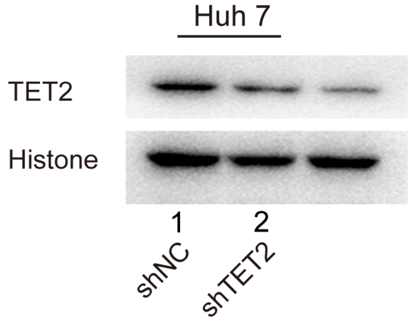

Full and uncropped western blot for Figure S3D,S3G&S3H

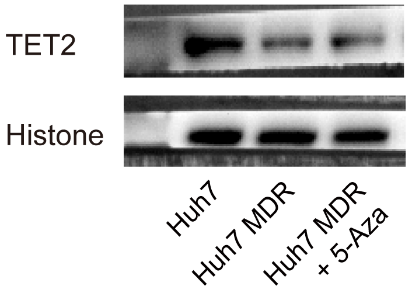

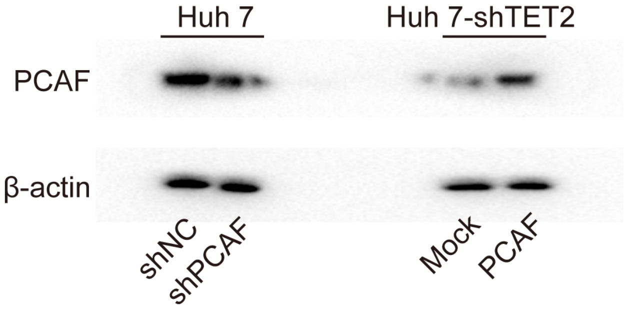

Full and uncropped western blot for Figure S3J

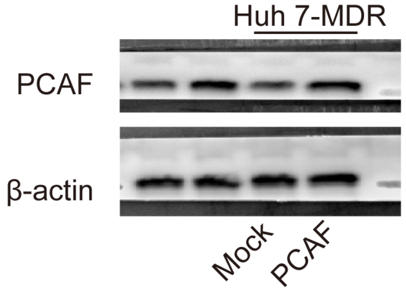

Full and uncropped western blot for FigureS4A

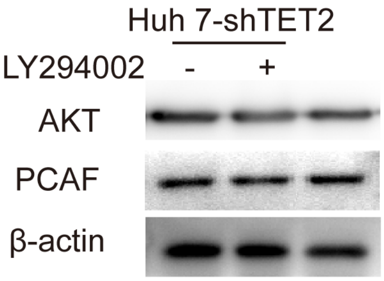

Full and uncropped western blot for FigureS4B&C

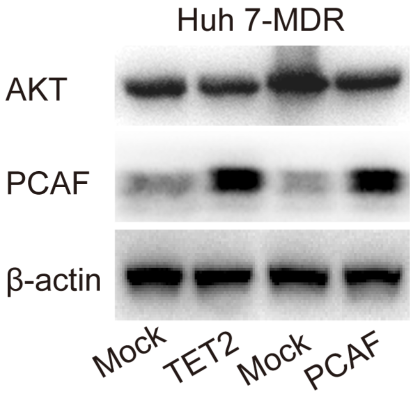

Supplement: Supplementary file 2 — Supplemental file_Uncropped WB [file 41419_2022_5406_MOESM2_ESM.pdf]
