# Supplementary figures and images for: Loss of 5-hydroxymethylcytosine induces chemotherapy resistance in hepatocellular carcinoma via the 5-hmC/PCAF/AKT axis
Source: Cell Death Dis. 2023 Feb 2;14(2):79. doi: 10.1038/s41419-022-05406-3 (PMC9895048; doi:10.1038/s41419-022-05406-3)

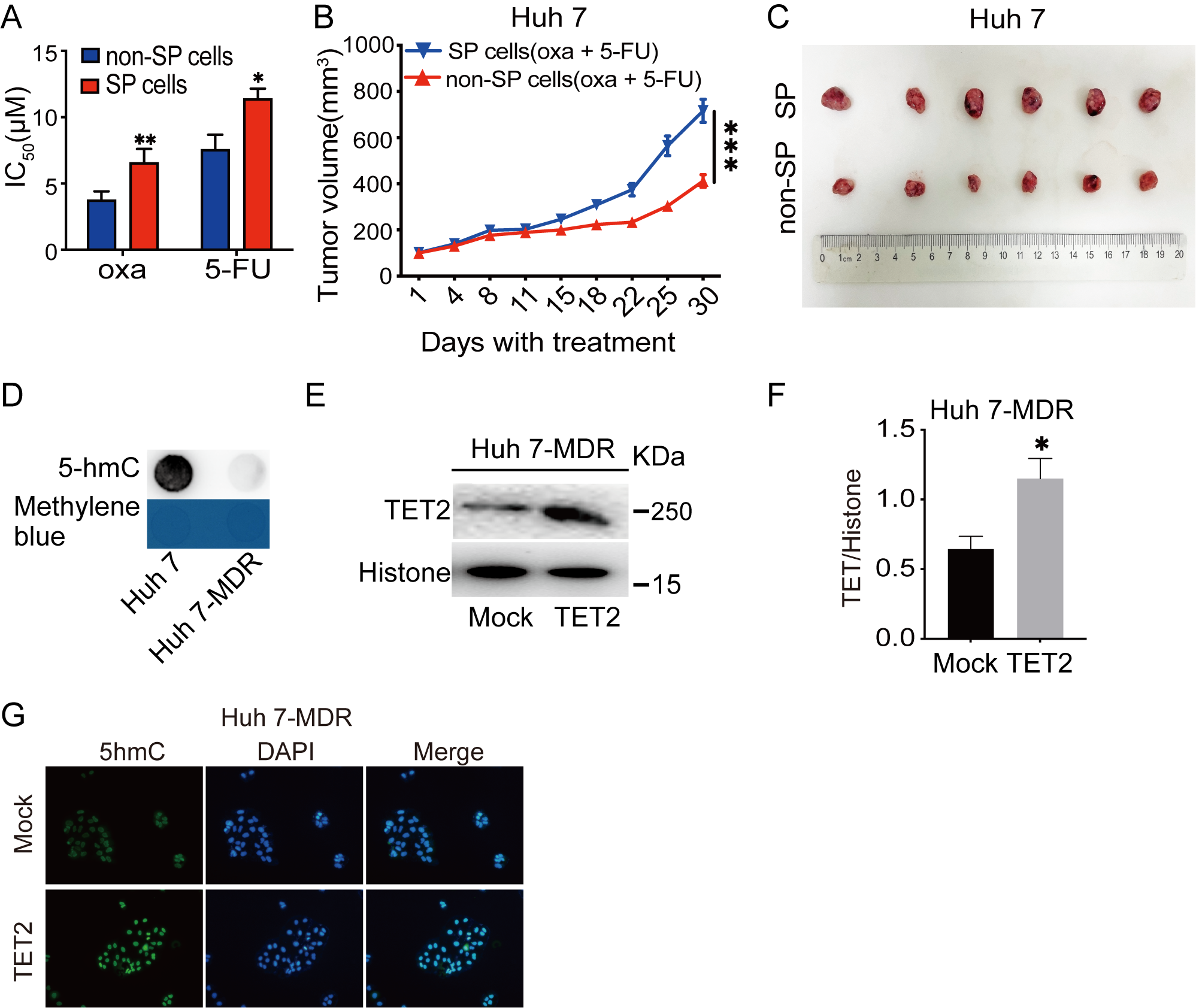

Supplement: Supplementary file 4 — Supplementary Figure 1 [file 41419_2022_5406_MOESM4_ESM.tif]

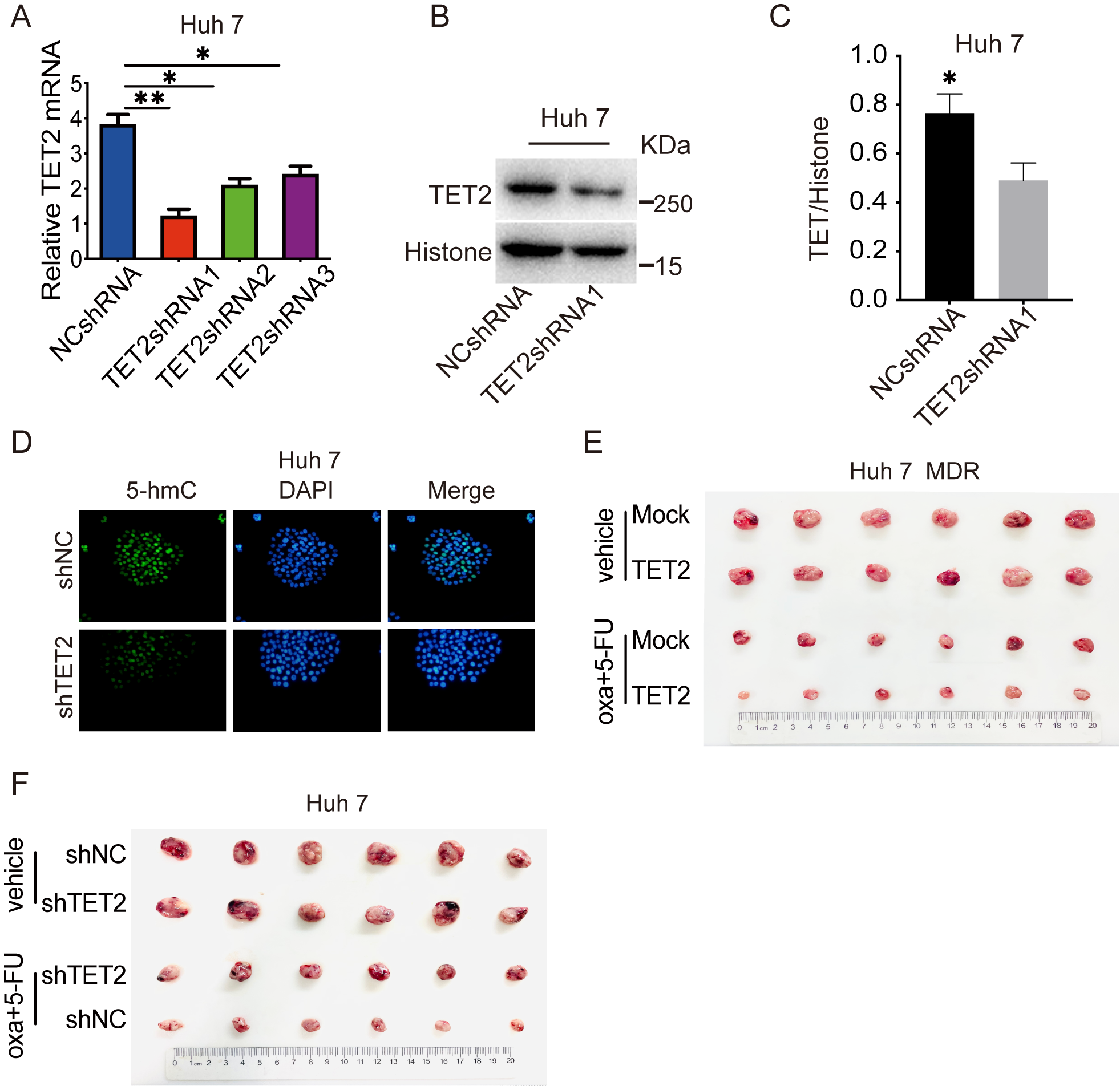

Supplement: Supplementary file 5 — Supplementary Figure 2 [file 41419_2022_5406_MOESM5_ESM.tif]

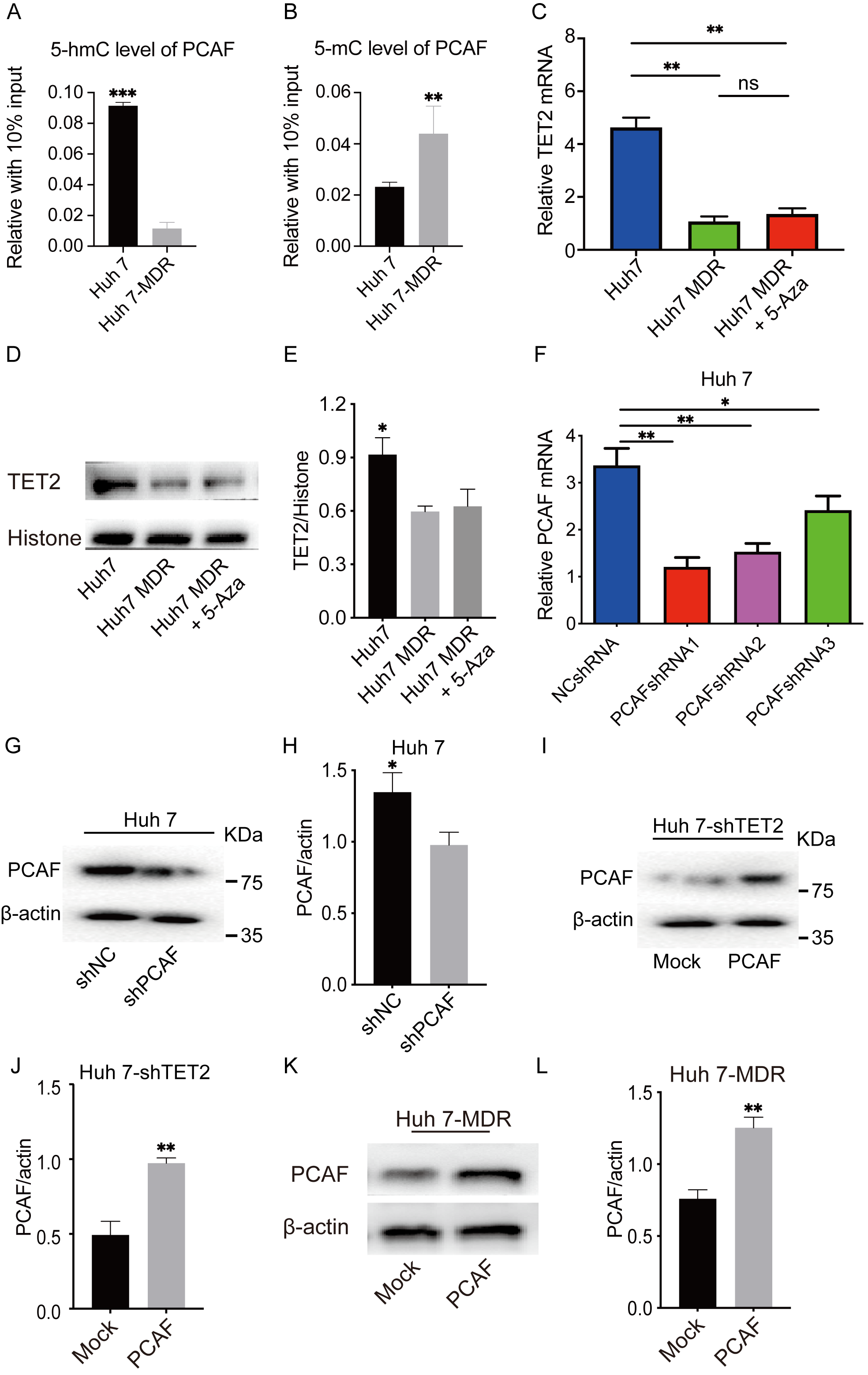

Supplement: Supplementary file 6 — Supplementary Figure 3 [file 41419_2022_5406_MOESM6_ESM.tif]

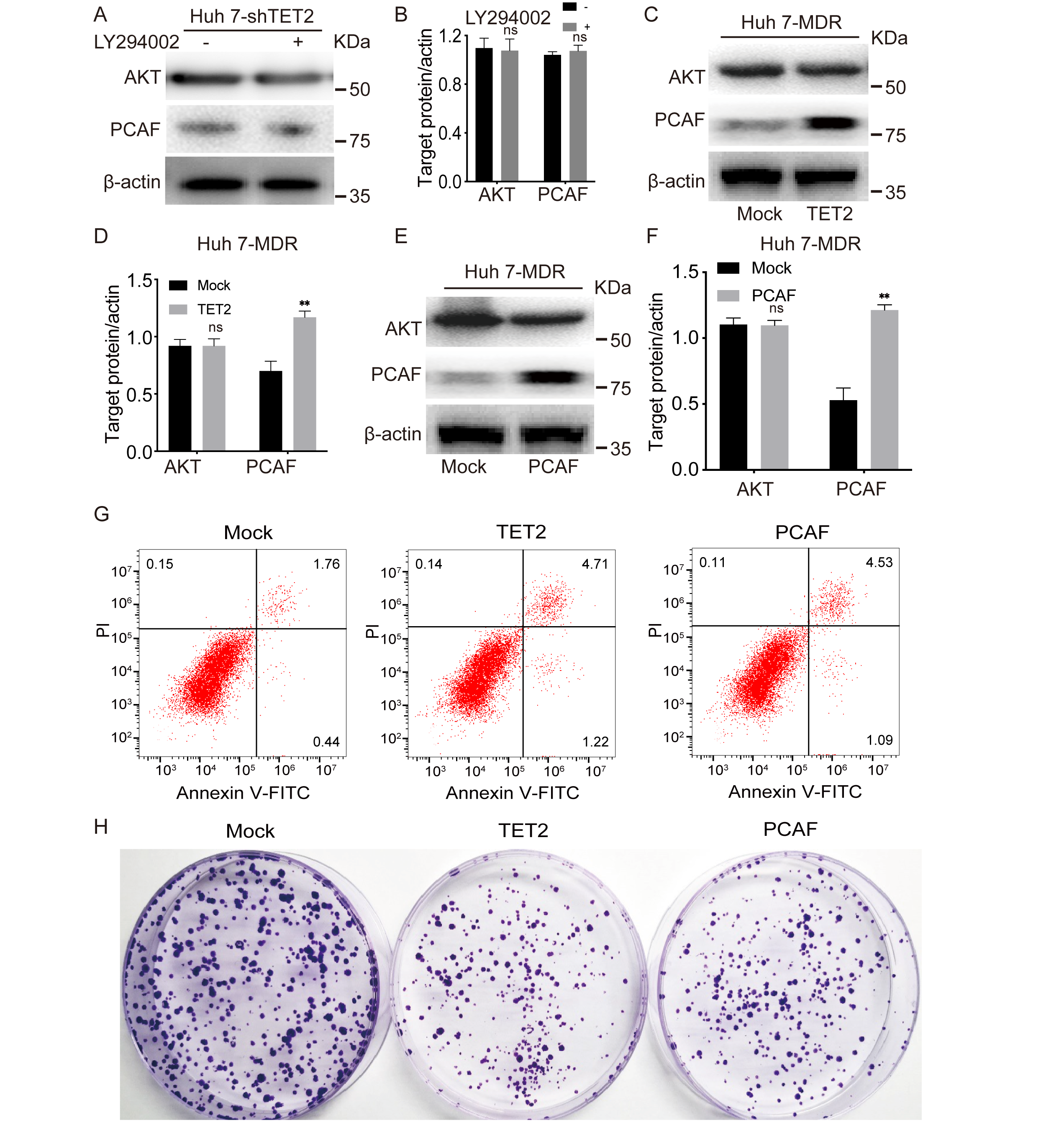

Supplement: Supplementary file 7 — Supplementary Figure 4 [file 41419_2022_5406_MOESM7_ESM.tif]
