# Supplementary material for: Loss of 5-hydroxymethylcytosine induces chemotherapy resistance in hepatocellular carcinoma via the 5-hmC/PCAF/AKT axis
Source: Cell Death Dis. 2023 Feb 2;14(2):79. doi: 10.1038/s41419-022-05406-3 (PMC9895048; doi:10.1038/s41419-022-05406-3)
Supplement: Supplementary file 8 — Supplementary Table 1 [file 41419_2022_5406_MOESM8_ESM.docx]

**Supplementary Table 1**. **Target sequences of TET2 and PCAF shRNA**

| **shRNA** | **Target sequence** |
| --- | --- |
| TET2 shRNA -1 | CTGCTGGATTGCTGCAAGG |
| TET2 shRNA -2 | CACACACATGGTGAACTCC |
| TET2 shRNA -3 | GGGTCGAGACAAGGAGCAA |
| PCAF shRNA -1 | CGAAGACTGCGATCTCCCA |
| PCAF shRNA -2 | CATTGCTTCGCTCGGTCTT |
| PCAF shRNA -3 | CCAGCCAGCTAGGCATCCA |
