# Supplementary material for: Loss of 5-hydroxymethylcytosine induces chemotherapy resistance in hepatocellular carcinoma via the 5-hmC/PCAF/AKT axis
Source: Cell Death Dis. 2023 Feb 2;14(2):79. doi: 10.1038/s41419-022-05406-3 (PMC9895048; doi:10.1038/s41419-022-05406-3)
Supplement: Supplementary file 9 — Supplementary Table 2 [file 41419_2022_5406_MOESM9_ESM.docx]

**Supplementary** **Table 2**. **The antibodies used in this study**

| **Antibody** | **Company** | **Cat No.** |
| --- | --- | --- |
| 5-mC | Abcam | ab10805 |
| 5-hmC | Abcam | ab231902 |
| TET1  TET2  TET3 | Abcam  Abcam  Abcam | ab191698  ab124297  ab153724 |
| PCAF | Abcam | ab12188 |
| AKT | Abcam | ab179463 |
| pAKT | Abcam | ab38449 |
| ERK | Abcam | ab54230 |
| pERK | Abcam | ab201015 |
| BCL2 | Abcam | ab32124 |
| BCL-XL | Abcam | ab32370 |
| BAX | Abcam | ab232479 |
| BCL-XS | eBioscience™ | 14-6994-81 |
| β-actin | Abcam | ab8226 |
| HRP-labeled Goat Anti-Rabbit IgG(H+L) | Beyotime | A0208 |
| HRP-labeled Goat Anti-mouse IgG(H+L) | Beyotime | A0216 |
| Alexa Fluor 488-labeled Goat Anti-Mouse IgG(H+L) | Beyotime | A0428 |
| Alexa Fluor 488-labeled Goat Anti-Rabbit IgG(H+L) | Beyotime | A0423 |
