# Supplementary material for: Loss of 5-hydroxymethylcytosine induces chemotherapy resistance in hepatocellular carcinoma via the 5-hmC/PCAF/AKT axis
Source: Cell Death Dis. 2023 Feb 2;14(2):79. doi: 10.1038/s41419-022-05406-3 (PMC9895048; doi:10.1038/s41419-022-05406-3)
Supplement: Supplementary file 10 — Supplementary Table 3 [file 41419_2022_5406_MOESM10_ESM.docx]

**Supplementary** **Table 3. Sequence of primers for RT-qPCR**

| **Gene** | **Forward primer (5’-3’)** | **Reverse primer (5’-3’)** |
| --- | --- | --- |
| TET2 | CCAGACAGAACCTCTGGCTAC | AAGGAGCCCAGAGAGAGAAGG |
| PCAF | GGAGGCACCATCTCAACGAAG | GGTTCTGGAAGAGGCTGAGAG |
| GAPDH | GGGGCTCTCCAGAACATCATCC | ACGCCTGCTTCACCACCTCTT |
